# Does Mixed Neuroendocrine-Nonneuroendocrine Neoplasm (MiNEN) of the Parathyroid Gland Exist? First Description of a Possible Case

**DOI:** 10.1007/s12105-020-01178-4

**Published:** 2020-06-06

**Authors:** Silvana Di Palma, Moonim Mufaddal, Vishwas Iyer, Amedeo Sciarra, Stefano La Rosa

**Affiliations:** 1 Department of Pathology, Royal Surrey Hospital, Guildford, UK; 2 Department of Pathology, St. Thomas Hospital, London, UK; 3 Institute of Pathology, Lausanne University Hospital and University of Lausanne, Lausanne, Switzerland; 4 Cellular Pathology Department, The Royal Surrey Hospital, Egerton Rd, Guildford, GU2 7XX UK

**Keywords:** Mixed neoplasm, Mixed neuroendocrine-nonneuroendocrine neoplasm, MiNEN, Composite tumor, Combined tumor, Parathyroid gland

## Abstract

We describe the histological, histochemical, and immunohistochemical features of an unusual neoplasm of the parathyroid gland showing the histologic criteria of a mixed neuroendocrine-nonneuroendocrine neoplasm (MiNEN). To the best of our knowledge, this is the first report of such a tumor. A 43-year old male presented with acute and severe abdominal pain due to acute pancreatitis. On physical examination a painless lump in the right neck was detected and laboratory analyses revealed hyperparathyroidism (parathormone: 146 pmol/L, normal range 1.05–6.83) and hypercalcemia (calcium level: 3.02 mg/dL, normal range 2.25–2.5), which fell to 2.55 mg/dL after parathyroidectomy. Histologically, the tumor was a parathyroid carcinoma associated with a mucous secreting adenocarcinoma also confirmed by histochemical (Alcian blue—periodic acid Schiff) and immunohistochemical stainings. The present case expands the spectrum of MiNENs that can be found in endocrine organs.

## Introduction

Mixed neuroendocrine-nonneuroendocrine neoplasms (MiNENs) are epithelial malignancies composed of both neuroendocrine and non-neuroendocrine components, which are morphologically and immunohistochemically recognizable, and constitute at least 30% of the tumor burden [1]. MiNENs should be distinguished from amphicrine carcinomas, which are composed of cells showing a divergent neuroendocrine and exocrine differentiation characterized by the simultaneous presence within the cytoplasm of the same cell of secretory granules and of mucous or exocrine antigens. Amphicrine carcinomas represent a peculiar entity whose clinico-pathologic, pathogenetic, molecular, and prognostic features are still to be elucidated [1].

MiNENs can virtually occur in any organ of the body and have been described in the pituitary gland, thyroid, nasal cavity, larynx, lung, digestive system, urinary system, male and female genital organs, and skin. However, no case has been identified in the parathyroid gland [2] and, for this reason, this entity was not included in the 2017 WHO classification of parathyroid tumors [1]. Despite extensive literature review, we have not found any case of parathyroid MiNEN. Here we report for the first time such a case, which expands the list of MiNENs that can be found in endocrine organs.

## Case Report

A 43-year old male presented at the Accident Emergency, Royal Surrey Hospital, Guildford, United Kingdom, with acute and severe abdominal pain due to acute pancreatitis. Previous history was unremarkable, except for the detection of kidney stones dating few months. On admission blood tests showed hypercalcemia (calcium level: 3.02 mg/dL, normal range 2.25–2.5 mg/dL) and high parathormone level (146 pmol/L, normal range 1.05–6.83 pmol/L) consistent with hyperparathyroidism. On examination, he had a painless lump in the right neck. Ultrasound and 99 m Tc-sestamibi scan revealed a solid and cystic tumor in the lower right parathyroid gland, 35 mm in diameter. With a clinical diagnosis of primary hyperparathyroidism, the patient underwent excision of the mass. Intraoperative assessment favored carcinoma for its large size (> 30 mm) and adherence to the esophagus and to the right laryngeal nerve. Postoperatively his calcium serum level fell to 2.55 mg/dL. Following the histological diagnosis, the patient underwent level VI neck dissection and received adjuvant radiotherapy. At his last follow-up, two year after surgery, he was well without evidence of recurrent disease. Then, patient moved to another region and was lost to follow up.

Part of this case was presented at slide seminar on endocrine pathology 30^th^ Congress of the European Society of Pathology, Bilbao September 2018. None of the attending endocrine pathologists had come across a similar case.

## Pathological Findings

The parathyroid gland was resected in two pieces, 45 g—45 × 25 × 15 mm and 3 g—20 × 9 × 7 mm. Formalin fixed paraffin embedded sections from both specimens were stained with Hematoxylin and Eosins (H&E) and Alcian Blue-Periodic Schiff (AB-PAS). Immunoreactions were automatically performed using the immunostainer Benchmark® XT ICH/ISH (Ventana, Tucson, CA, USA) using the antibodies listed in Table 1.

**Table 1:** Antibodies and antisera used

Antibody	Dilution	P/M (Clone)	Source
Synaptophysin	1:100	M (snp88)	BioGenex Laboratories, San Ramon, CA, USA
Chromogranin A	1:1	M (LK2H10)	Ventana Medical System, Tucson, AZ, USA
Parathormone	1:40	P	BioGenex Laboratories
Parafibromin	1:40	M (2HI)	Santa Cruz Biotechnology, Santa Cruz, CA, USA
GATA3	1:50	M (L50-823)	Biocare Medical, Pacheco, CA, USA
Pan-cytokeratin	1:100	M (AE1/AE3)	Dako, Copenhagen, Denmark
Cytokeratin 7	1:200	M (O-TL 12/30)	Dako
Thyroglobulin	1:600	M (ID4)	Leica Biosystem Newcastle Ltd, New Castle, UK
TTF1	1:2	M (8G7G3/1)	NeoMarkers, Fremont, CA, USA
Calcitonin	1:1	P	Signet, Dedham, MA, USA
Glucagon	1:2500	P	Milab, Malmo, Sweden
p53	1:500	M (D07)	Dako
S100	1:400	P	Leica Biosystem Newcastle Ltd
PGP-9.5	1:100	M (13C4)	Biomeda Corporation, Foster City, CA
Bcl-2	1:1	M (790–4604)	Ventana Medical System
Ki67	1:100	M (MIB1)	Dako

*P/M* polyclonal/monoclonal, *RTU* ready to use

**Fig. 1:**
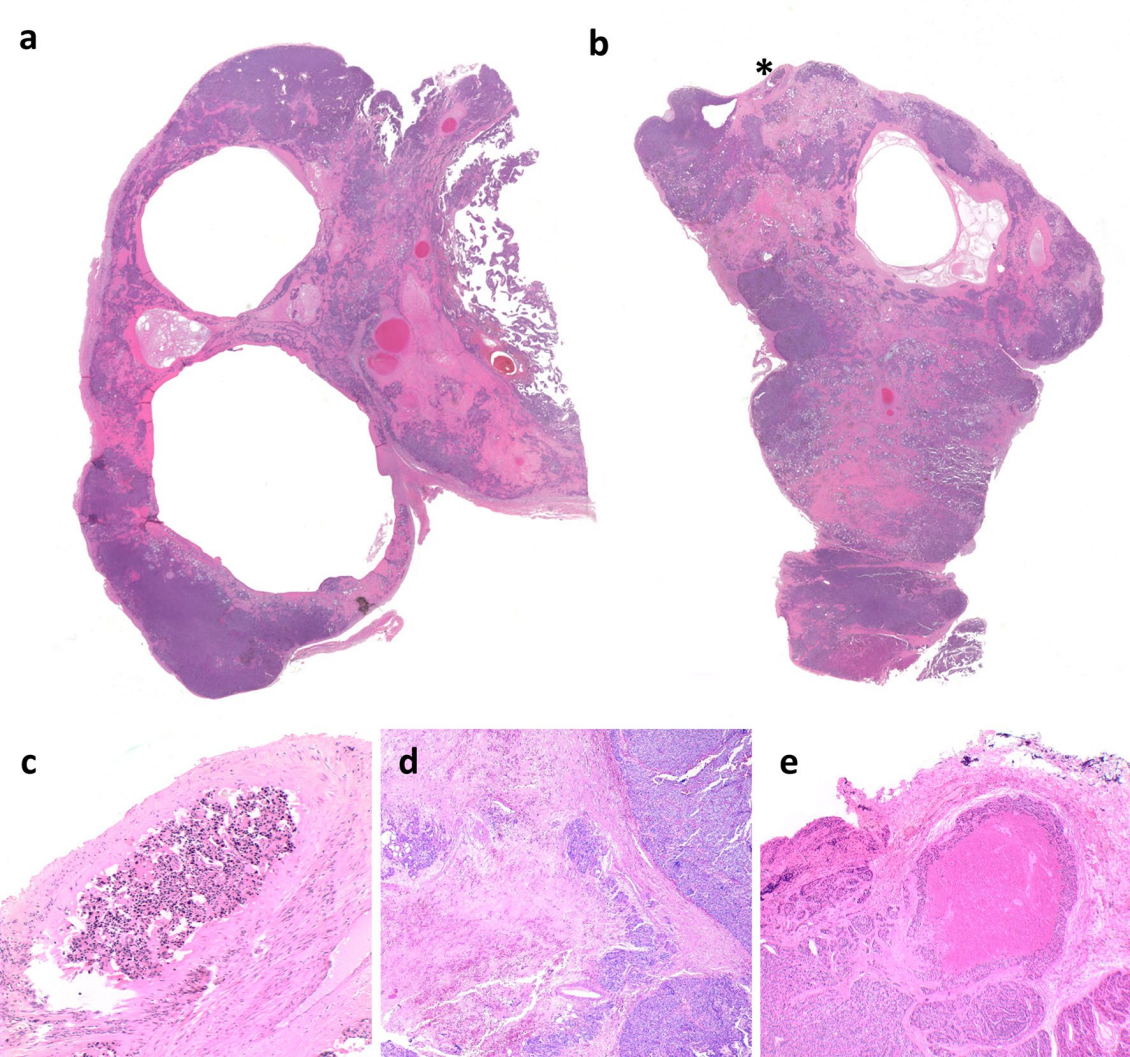
The parathyroid neoplasm was received in two pieces and shows a solid and cystic appearance with fibrous bands (**a**, **b**). Asterisk Shows tumor cells in a vessel, better visualized in c. The neuroendocrine component of the tumor infiltrates the capsule with extension in the surrounding connective tissue (**c**). Necrosis is also observed (**d**) (**a**, original magnification × 200; **c**–**e**, original magnification × 100)

**Fig. 2:**
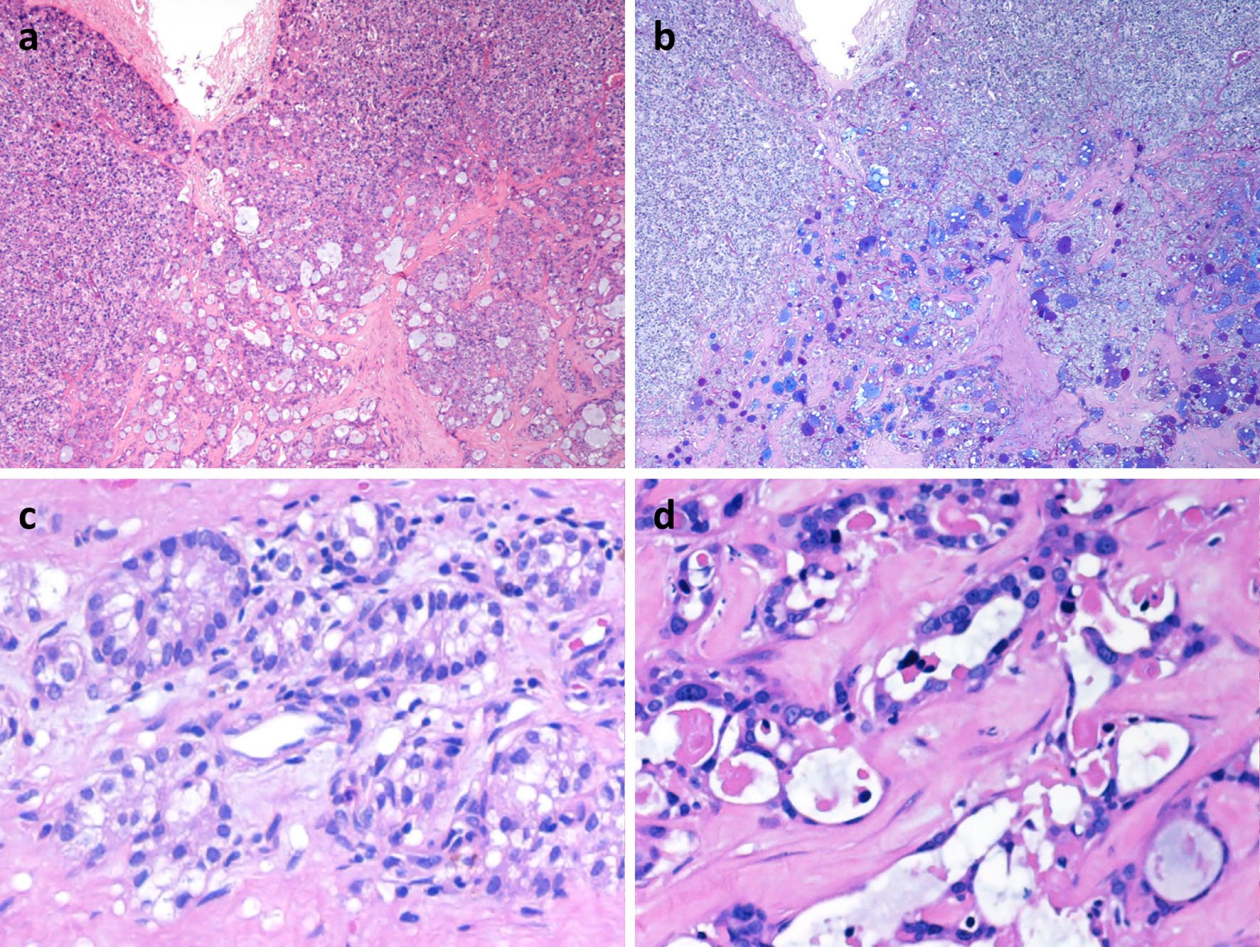
The tumor is composed of two different neoplastic populations: a neuroendocrine solid component (**a**, **b**, upper) and an adenocarcinomatous component (**a**, **b**, bottom). The first component was composed of chief cells arranged in nested, trabecular, lobular, and solid patterns separated by dense fibrous bands (**c**). The adenocarcinoma component was composed of cuboidal cells arranged in glandular architecture with small sized tubules (**d**) containing AB-PAS positive mucin embedded in dense fibrous tissue (**b**). (**a**, **b**, original magnification × 100; **b**, **c**, original magnification × 400)

**Fig. 3:**
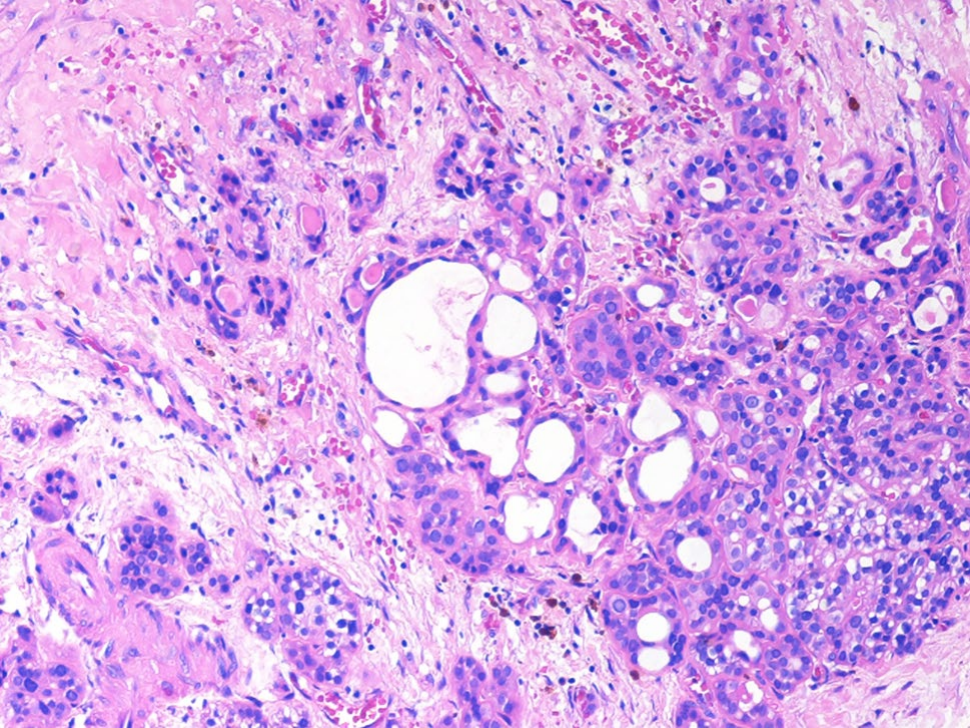
The two tumor components were partially intermingled. In this image, the parathyroid neoplasm is well evident at the bottom right, while the glandular component is observed in the upper part (original magnification × 200)

At low power magnification, the tumor appears well circumscribed, solid and cystic (Fig. 1a and 1b). It was composed of two different, partially intermingled, neoplastic populations (Figs. 2a, 3). A major solid “neuroendocrine-looking” component, representing about 60% of the tumor, was admixed with another component arranged in glandular architecture (adenocarcinoma-like). The histology of the predominant component was congruent with that of a conventional parathyroid neoplasm, showing chief cells arranged in nested, trabecular, lobular, and solid patterns separated by dense fibrous bands (Fig. 2c). Varying proportion of oxyphilic cells were also seen. In about 10% of neuroendocrine tumor cells nuclear pleomorphism was observed and interpreted as “endocrine atypia”. The mitotic count reached five mitoses × 10 HPF (× 400). The presence of vascular invasion, infiltration of the tumor capsule with extension into the adjacent connective tissue, and necrosis (Fig. 1c–e) led us to the diagnosis of parathyroid carcinoma. The minor component was composed of cuboidal cells arranged in glandular architecture with small sized tubules containing AB-PAS positive mucin embedded in dense fibrous tissue (Fig. 2b, d). No lymph node metastases were found.

**Fig. 4:**
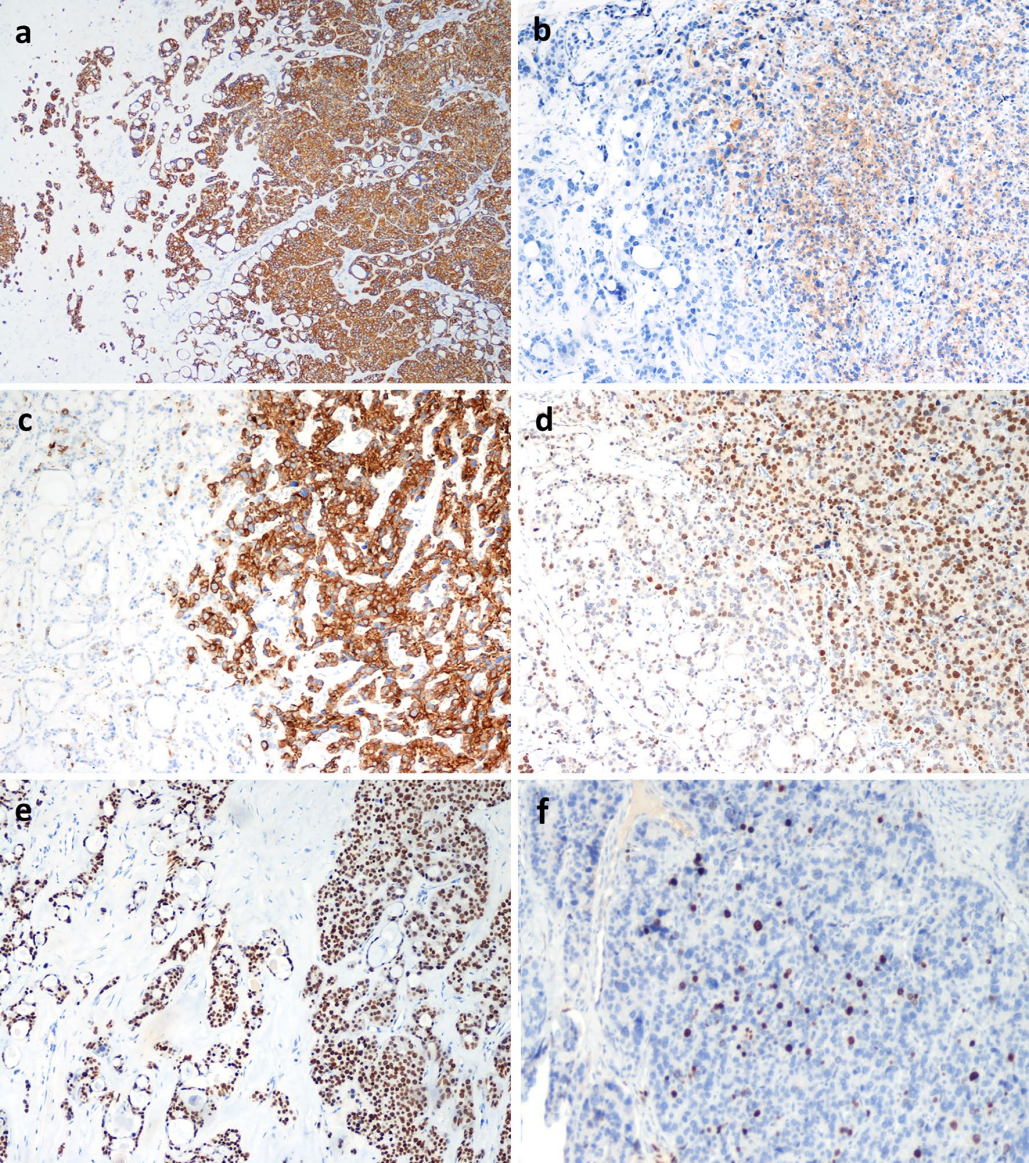
Neoplastic cells of both components are positive for CAM 5.2 (**a**), while chromogranin A is restricted to the solid neuroendocrine component (**b**), which is also positive for parathormone (**c**) and parafibromin (**d**). GATA 3 is positive in both components (**e**). The Ki67-proliferative index in the adenocarcinoma and neuroendocrine component is 10% (**f**) (**a**–**e** original magnification × 100, **f**  × 200)

**Fig. 5:**
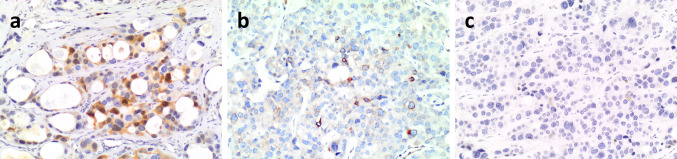
The neuroendocrine component contains cells positive for PGP9.5 (**a**) and scattered cells were also positive for bcl-2 (**b**). p53 was completely negative in tumor cells of the both components (**c**). (**a**–**c**, original magnification × 200)

**Fig. 6:**
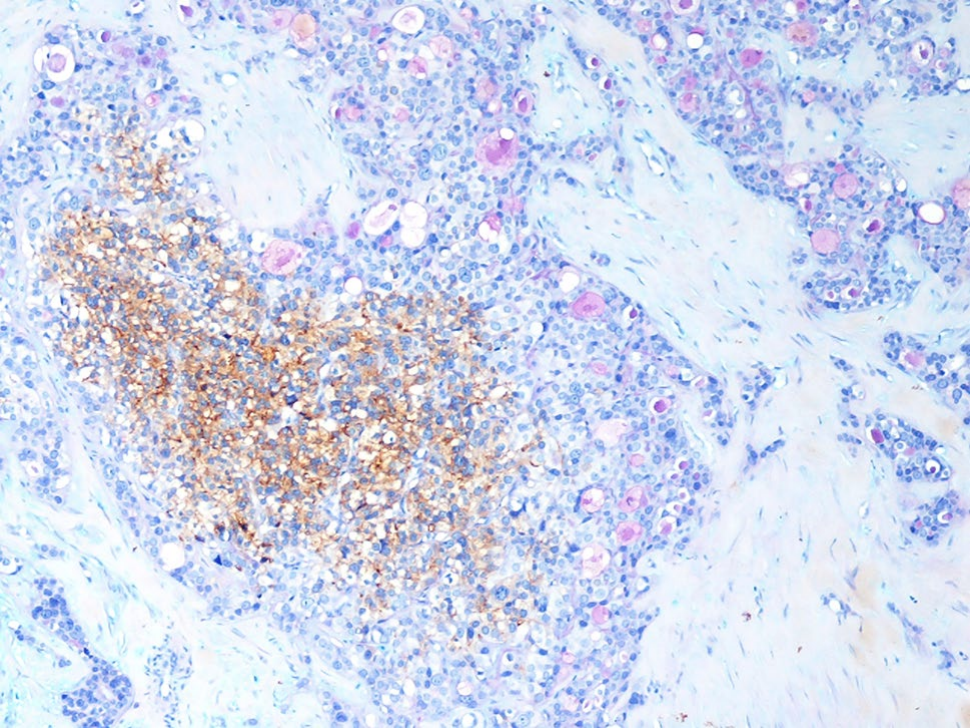
Double immunohistochemical stain for chromogranin A and AB-PAS stain demonstrates that only the neuroendocrine solid component is chromogranin A-positive, while the adenocarcinoma component produces mucous (original magnification × 200)

Most tumor cells of the neuroendocrine component were immunoreactive for CAM.5.2, cytokeratin (CK) 7, CK AE1/AE3, synaptophysin, chromogranin A, parathormone, GATA3, and parafibromin (Fig. 4), whilst they were negative for thyroglobulin, TTF1, calcitonin, glucagon, and S100. PGP9.5 and Bcl*-2* were positive in a minority of cells (Fig. 5a, b). Ki67 proliferative index, evaluated by counting the number of positive cells in at least 500 tumor cells in hot spot areas on camera-captured printed images, was 10%. Neuroendocrine markers were expressed in the neuroendocrine-looking area, sparing the tubulo-glandular component. Conversely, AB-PAS stain was restricted to the adenocarcinoma-like area (Fig. 6). No evidence of amphicrine cells (i.e. expressing both chromogranin A and AB-PAS positive mucous in the cytoplasm) was detected upon careful examination double stained sections. Cells of the adenocarcinoma component were CAM.5.2, CK7, CK AE1/AE3, and GATA-3 positive and negative for synaptophysin, chromogranin A, parathormone, parafibromin (Fig. 4), thyroglobulin, TTF1, calcitonin, glucagon, S100, PGP-9 and Bcl-2. The Ki67 proliferative index in the adenocarcinoma component was 10%. p53 was uniformly and completely negative in tumor cells of both components (Fig. 5c), suggesting *TP53* mutation [3, 4].

## Discussion

Histological, histochemical and immunohistochemical studies of this parathyroid neoplasm confirmed the presence of two different tumor components: a neuroendocrine one, meeting the criteria for parathyroid carcinoma, and a non-neuroendocrine component, meeting the criteria for a mucous-secreting adenocarcinoma. The mutually exclusive expression of neuroendocrine markers and of histochemical evidence of intracellular and extracellular mucous has provided additional support for the diagnosis of a *mixed type* tumor. The coexistence of neuroendocrine and non-neuroendocrine components in the same epithelial neoplasm has virtually been described in all endocrine and non-endocrine organs, but to date no case was reported in the parathyroid gland. Indeed, the 2017 WHO classification of parathyroid tumors includes typical and atypical parathyroid adenoma and carcinoma, with no mention of mixed variants as a specific tumor entity [1]. MiNENs are more frequent in the digestive system, where they represent a heterogeneous group of neoplasms in which both the non-neuroendocrine and neuroendocrine components can have different morphological features including, for the former, adenocarcinomas or squamous cell carcinomas with different degrees of differentiation and, for the latter, well differentiated or poorly differentiated neuroendocrine neoplasms [5]. The different possible combinations of these two components give rise to different entities showing peculiar morphological, immunohistochemical, molecular, and clinical features [2]. It has been demonstrated that both components of MiNENs have a common origin from a precursor progenitor stem cell, which undergoes divergent differentiation [6–10]. A similar pathogenesis has also been proposed for pituitary and nasal MiNENs [11, 12]. In the thyroid gland, MiNENs composed of medullary thyroid carcinoma and either of follicular or papillary carcinoma have been described [1] but, conversely to digestive MiNENs, they are considered as tumors arising from two distinct (follicular and para-follicular) cell types [13]. In addition, rarer cases of mucinous variant of follicular and oncocytic carcinoma of the thyroid have been reported [14, 15] and these interesting neoplasms show some similarity with our case since they were able to produce mucous.

The present case presents at least three issues for discussion, which deserve to be pointed out. First, the correct classification of the current tumor proved to be difficult as no other similar cases were found in the literature. Given the parathyroid site of origin, we could not apply the same diagnostic criteria used for the digestive system neither we could use the 2017 WHO classification of tumors of parathyroid gland which only includes parathyroid adenoma and carcinoma [1]. Although in a recently published comprehensive review of the literature parathyroid MiNENs were not described [2], we believe that the present case may be classified as MiNEN, so adding the spectrum of possible sites of origin of these peculiar mixed neoplasms. Indeed, despite a well circumscribed appearance at low power magnification, the presence of vascular invasion together with infiltration of the surrounding fibrous tissue led us to diagnose the neuroendocrine component of the neoplasm as parathyroid carcinoma. The presence of fibrous bands, necrosis and a Ki67 proliferative index of 10%, although no specific criteria of malignancy if considered alone, indirectly supported this diagnosis in the presence of the other two accepted morphological criteria of malignancy (vascular invasion and infiltration of the capsule). Because the parathyroid carcinoma was associated with an adenocarcinoma, we have thought that the term MiNEN was the more appropriate to define this unique and never previously described parathyroid neoplasm. The panel of immunohistochemical stainings, the clinical history, the lack of any elsewhere located neoplasms after careful imaging investigation, and the two years of follow-up have supported our hypothesis of the primary and not metastatic origin of the adenocarcinoma component.

Second, the prognostic meaning of the present case remains to be determined. The prognostic role of Ki67-proliferative index, well defined in gastrointestinal MiNENs [16], is unknown in our case. The Ki67-proliferative index has been proposed as marker of malignancy in parathyroid neoplasms, but due to the overlapping counts between adenomas and carcinomas it should be used with caution [1]. Moreover, the prognostic role of Ki67 index in parathyroid carcinoma remains to be elucidate as well [1].

Third, the pathogenesis of this neoplasm needs to be clarified and, in particular, if the two components derive either from independent different stem cells, which give rise to two different neoplasms, or from a single stem cell undergoing divergent differentiation. Most of available molecular studies on the origin and development of MiNENs, mainly performed in digestive cases composed of adenocarcinoma and NEC (MANEC), demonstrated that the two components derive from a single precursor cell, which undergoes divergent differentiation after the first tumorigenic steps [6–8]. A similar finding was also observed in a nasal MiNEN composed of mucinous adenocarcinoma and NEC [12]. Mechanisms underlying the pathogenesis and the development of MiNENs composed of adenocarcinoma and NET, which may correspond to the present parathyroid MiNEN, have been less investigated. However, available data on lung and digestive cases demonstrated a clonal relationship between the two components as well [9, 10]. A similar feature was also observed in mixed neoplasms of the digestive system composed of adenoma and NET (MANET), although they are not considered MiNENs by definition [17]. The close morphologic relationships between the two components of our case (Figs. 2a, 3, and 6) suggests a common origin of the two components as also observed in a rare pituitary MiNEN composed of pituitary neuroendocrine tumor and craniopharyngioma [11]. Why a precursor cell may differentiate in this specific location into an adenocarcinoma is not known and unclear. This may be related to the specific embryologic origin, but further molecular studies on microdissected areas of the tumor may be useful to finally solve this issue.
